# Effects of swimming intervention on motor competence and dynamic balance in children at risk for developmental coordination disorder: a pre-post intervention study

**DOI:** 10.3389/fpsyg.2026.1815930

**Published:** 2026-05-04

**Authors:** TianYue Zhao, Huan Feng, XiaoPeng Hou, Yanting Li, Xiaojun Wang

**Affiliations:** 1 School of Physical Education, Sichuan University, Chengdu, Sichuan, China; 2 Chengdu Sport University, Chengdu, Sichuan, China

**Keywords:** developmental coordination disorder, dynamic balance, MABC-2, motor competence, swimming intervention, Y-balance test

## Abstract

**Background:**

Developmental Coordination Disorder (DCD) affects approximately 5–6% of school-aged children and is characterized by significant motor skill impairments that may persist into adulthood without intervention. Aquatic-based swimming programs have been proposed as a feasible approach to support motor development in children with neurodevelopmental motor difficulties. However, evidence specifically evaluating structured swimming interventions for DCD-related motor impairments remains limited.

**Objective:**

To describe pre-post changes in motor competence and dynamic balance following a structured swimming intervention in children at risk for DCD.

**Methods:**

Fourteen children at risk for DCD (9 boys, 5 girls; mean age 8.25 ± 0.83 years) participated in a single-group pre-post intervention study involving a 10-week breaststroke swimming program (20 sessions, 60 min each, twice weekly). Motor competence and dynamic balance were assessed before and immediately after the intervention. Motor competence was assessed using the Movement Assessment Battery for Children-Second Edition (MABC-2), and dynamic balance was evaluated with the Y-Balance Test Lower Quarter (YBT-LQ) at the pre- and post-intervention time points.

**Results:**

MABC-2 Total Test Score increased from pre- to post-intervention (median change 5.50; unadjusted *p =* 0.007, adjusted *p =* 0.021; r = 0.72), and Aiming and Catching showed a positive pre-post change (median change 25.00; unadjusted *p =* 0.004, adjusted *p =* 0.014; r = 0.77). In the YBT-LQ, five of six reach directions were higher at post-intervention than at baseline at the unadjusted level, and four directions remained significant after Holm correction, with medium-to-large effect sizes (Cohen’s dz. = 0.77–0.85). The intervention achieved a 100% retention rate with no adverse events.

**Conclusion:**

In this single-group pre-post study, children at risk for DCD showed positive pre-post changes in motor competence and dynamic balance over the 10-week study period, with the clearest changes observed in overall MABC-2 performance, aiming and catching, and several YBT-LQ reach directions. These findings provide preliminary, feasibility-informed evidence under structured conditions and suggest that this intervention model may warrant further controlled evaluation. However, studies including control conditions and functional outcome measures are needed before broader practice recommendations can be made for children at risk for DCD.

## 1 Introduction

Developmental Coordination Disorder (DCD) is a prevalent neurodevelopmental condition affecting approximately 5–6% of school-aged children and is characterized by substantial difficulties in acquiring and executing coordinated motor skills that interfere with daily activities and academic achievement (Zwicker et al., 2012; Blank et al., 2019). Children with DCD typically present with clumsiness, slowness, and inaccuracy in age-appropriate motor tasks (Biotteau et al., 2016a; Wilson et al., 2017), and these motor difficulties are often accompanied by reduced physical activity participation, poorer health-related fitness, and psychosocial challenges (Rivilis et al., 2011; Missiuna et al., 2014). Because motor difficulties frequently persist over time without effective support (Cantell et al., 2003; Kirby et al., 2008), identifying feasible movement-based interventions remains an important clinical and educational priority.

Motor competence in DCD is commonly assessed using the Movement Assessment Battery for Children (MABC), which captures manual dexterity, aiming and catching, and balance, and has shown good reliability across populations (Henderson et al., 1992; Ellinoudis et al., 2011; Smits-Engelsman et al., 2018). Dynamic balance can be further evaluated using the Y-Balance Test Lower Quarter (YBT-LQ), which also demonstrates acceptable test–retest reliability in children (Butler et al., 2013; Shaffer et al., 2013; Faigenbaum et al., 2014).

Current intervention approaches for DCD increasingly emphasize task-oriented and motor-based practice (Smits-Engelsman et al., 2013; Miyahara et al., 2017). Within this broader context, aquatic exercise has been proposed as a potentially useful training environment because buoyancy reduces body-weight demands, while water resistance and hydrostatic pressure provide continuous sensory input during movement (Kelly and Darrah, 2005; Getz et al., 2006; Fragala-Pinkham et al., 2008; Becker, 2009). These characteristics may be particularly relevant for children with coordination difficulties, as they may support repeated whole-body practice under conditions that are perceived as safer and less physically demanding.

Swimming programs have been associated with favorable motor competence outcomes in typically developing children (Lopes et al., 2012; Moura et al., 2021). In pediatric neurodevelopmental populations more broadly, aquatic therapy has also been associated with positive changes in gross motor function and balance (Dimitrijević et al., 2012; Shariat et al., 2024; Carson et al., 2025). Nevertheless, this broader literature cannot be assumed to translate directly to DCD. Evidence specifically evaluating structured swimming interventions for children at risk for DCD remains scarce, and the few available studies are limited by short duration, small samples, or heterogeneous intervention content (Hillier et al., 2010). As a result, it remains unclear whether a standardized swimming program is associated with measurable pre-post changes in motor competence and dynamic balance in this population, and whether the proposed benefits of the aquatic environment are sufficient to support measurable pre-post change (Missiuna et al., 2007; Adams et al., 2014).

Accordingly, this study examined pre-post changes in motor competence and dynamic balance over a 10-week breaststroke swimming program in children at risk for DCD. Accordingly, this study investigated pre-post alterations in motor proficiency and dynamic equilibrium throughout a 10-week breaststroke swimming intervention for children with potential DCD. The research sought to address the following inquiries: (RQ1) Were there significant pre-post modifications in motor competence (as measured by MABC-2 Total Test Score and subdomain scores) during the 10-week investigation period for children at risk for DCD? (RQ2) Were pre-post variations detected in dynamic balance capabilities (YBT-LQ reach distances across multiple directions and both extremities) over the identical timeframe? Employing an exploratory single-group methodology, this research aimed to characterize both the direction and extent of pre-post change in both MABC-2 and YBT-LQ outcomes throughout the intervention period.

## 2 Methods

### 2.1 Study design

This study employed a single-group pre-post intervention design to examine within-participant pre-post changes in motor competence and dynamic balance following a structured swimming program in children at risk for DCD. Because no control group was included, the study was positioned as an exploratory intervention study intended to characterize change over time and feasibility rather than to establish causal efficacy. All participants received the same 10-week swimming intervention, with outcome assessments conducted at two time points: baseline (T0, pre-intervention) and immediately following the 10-week program (T1, post-intervention). The study, including participant recruitment, screening, intervention delivery, and post-intervention assessment, was conducted in Chengdu, China, between 22 September 2025 and 3 January 2026. Participant recruitment and screening took place from 22 September 2025 to 16 October 2025, and the intervention together with post-intervention assessments was completed between 25 October 2025 and 3 January 2026. The study design and procedures were reviewed and approved by the Ethics Committee of Chengdu Sport University (Approval No.: CDSU-Ethics [2025] 205) and conducted in accordance with the Declaration of Helsinki. Written informed consent was obtained from all participants’ legal guardians, and verbal assent was obtained from all child participants prior to study enrollment. All participants were informed of their right to withdraw from the study at any time without penalty. Personal data were anonymized to protect participant privacy. Assessors conducting the MABC-2 and YBT-LQ evaluations were blinded to the study hypotheses and participants’ baseline performance scores during post-intervention assessments. Because of the behavioral nature of the intervention, participant and coach blinding was not feasible.

### 2.2 Sample size

Sample size calculation was performed using G*Power software (version 3.1.9.7, Heinrich-Heine-Universität Düsseldorf, Düsseldorf, Germany) (Kang and Huh, 2021). Small samples are not uncommon in preliminary intervention research on developmental coordination disorder (DCD). Previous DCD intervention studies have included 8 children in a repeated-measures goal-orientated group intervention, 13 children in an aquatic pilot randomized controlled trial, 22 children in a randomized controlled pilot study, and 24 children across two pilot group-intervention programs (Hillier et al., 2010; Dunford, 2011; Au et al., 2014; Caçola et al., 2016). In addition, a systematic review of high-quality randomized controlled trials in DCD reported a median sample size of 28.5, with a range of 13 to 58 participants across nine trials (Preston et al., 2017). Taken together, these findings suggest that relatively small samples are common in early-phase DCD intervention studies, particularly when the design is pilot, feasibility-oriented, or based on within-subject pre-post comparisons. For the present study, the *a priori* calculation was based on the primary pre-post comparison in a paired-samples design, with the MABC-2 Total Test Score specified as the principal motor outcome. Previous meta-analyses examining motor-based interventions for DCD have reported large effect sizes for task-oriented approaches, with Cohen’s d ranging from 0.83 to 1.06. Considering these findings and the unique proprioceptive and neuromuscular benefits of aquatic environments for motor skill development (Becker, 2009), we anticipated a large effect size (Cohen’s dz. = 0.85) for the swimming intervention. With alpha set at 0.05 (two-tailed) and statistical power at 0.80 for a paired t-test, the a priori analysis indicated that a minimum sample size of 13 participants was required. To account for an anticipated dropout rate of approximately 15–20%, we aimed to recruit 15–16 participants (i.e., 13/(1–0.15) ≈ 16; 13/(1–0.20) ≈ 17). Ultimately, 14 eligible children were enrolled, and all participants completed both pre- and post-intervention assessments (retention rate 100%). Under the assumed effect size (dz = 0.85), the achieved power with *n =* 14 was approximately 0.84, indicating adequate statistical power for the planned analyses.

### 2.3 Participants

Participants were recruited from Grade 1 to Grade 3 students (*n =* 234; 130 boys, 104 girls) at Xihanggang Primary School in Shuangliu District, Chengdu, Sichuan Province, China. A two-stage screening procedure was used. In Stage 1, classroom teachers identified children who showed persistent motor difficulties during daily school activities or physical education classes on the basis of routine teacher observations, academic functioning in the school setting, and school health records. This pragmatic pre-screening step was used to reduce the number of children requiring formal motor testing and was not intended to serve as a clinical diagnosis. Children with obvious intellectual disability, sensory impairment, or acute medical conditions contraindicating physical activity were excluded at this stage, resulting in 90 candidates for formal assessment.

Following school approval and written informed consent from legal guardians (with child verbal assent), all pre-screened candidates underwent standardized assessment using the Movement Assessment Battery for Children-Second Edition (MABC-2). Children scoring below the 16th percentile were classified as being at risk for DCD on the basis of motor performance and were eligible only if they belonged to MABC-2 Age Band 2 (7–10 years). Because participant classification was based on MABC-2 motor performance alone, without a formal DSM-5 diagnostic process, the present sample should be interpreted as children at risk for DCD rather than children with a confirmed clinical diagnosis of DCD. In addition, no separate standardized measure was used to document DSM-5 Criterion B, namely interference with activities of daily living, school productivity, or participation.

Baseline characteristics are presented in Table 1. Fourteen children (mean age = 8.25 ± 0.83 years; 9 boys, 5 girls) met the at-risk criterion and participated in the intervention. Notably, the proportion of children classified as at risk for DCD (14/234 = 5.98%) is broadly consistent with epidemiological estimates (approximately 5–6%), supporting the plausibility of the school-based screening outcome. The sex distribution (9 boys, 5 girls) is consistent with epidemiological evidence indicating a higher prevalence of DCD-related motor difficulties in boys than in girls.

**Table 1:** Baseline demographic and anthropometric characteristics (*N =* 14).

Variable	Mean ± SD	Range
Age (years)	8.25 ± 0.83	7.0–9.5
Sex (male/female), n (%)	9 (64.3)/5 (35.7)	–
Leg length (cm)		
Left	63.57 ± 4.94	56.0–75.0
Right	63.50 ± 5.00	56.0–75.0
Arm length (cm)		
Left	39.64 ± 3.75	34.0–47.0
Right	39.64 ± 3.75	34.0–47.0

Data are presented as mean ± standard deviation (SD) for continuous variables and *n* (%) for categorical variables. All measurements were taken at baseline. Leg/arm length was measured to normalize YBT-LQ reach distances.

*Inclusion criteria*: (1) MABC-2 scores below the 16th percentile; (2) aged 7–10 years, corresponding to MABC-2 Age Band 2; (3) no prior structured intervention specifically targeting motor coordination during the recruitment period; (4) written informed consent from a parent or legal guardian, together with child assent; (5) medical clearance for aquatic activities.

**Figure 1:**
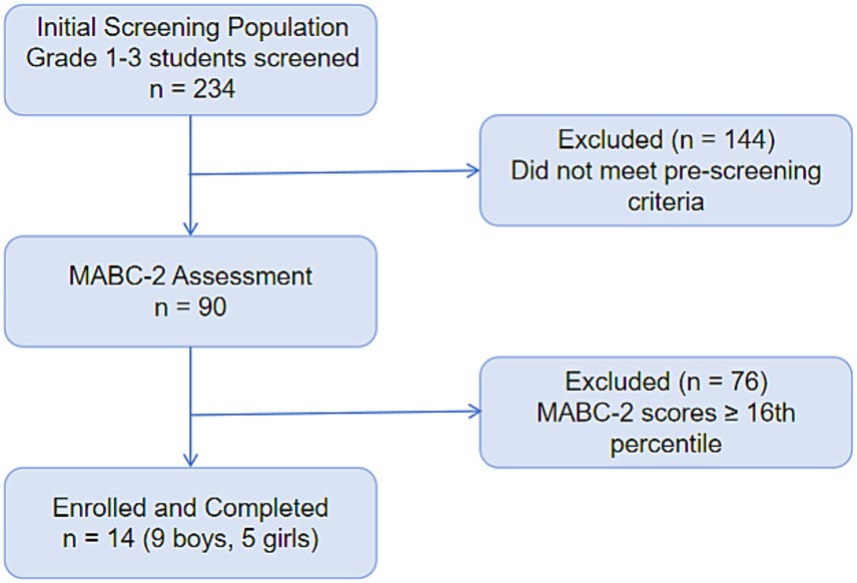
Participant recruitment and screening flowchart.

*Exclusion criteria*: (1) diagnosed neurological or movement disorders documented in school health records or reported by parents; (2) musculoskeletal injury or cardiopulmonary condition contraindicating swimming participation; (3) concurrent therapy or rehabilitation programs likely to influence motor outcomes; (4) inability to understand simple instructions or to attend the scheduled sessions regularly; (5) prior systematic swimming training. The detailed participant recruitment and screening process is illustrated in Figure 1.

Accordingly, the cohort should be regarded as a school-based at-risk sample identified through standardized motor screening rather than as a clinic-referred sample with a confirmed DSM-5 diagnosis of DCD.

### 2.4 Swimming intervention protocol

**Figure 2:**
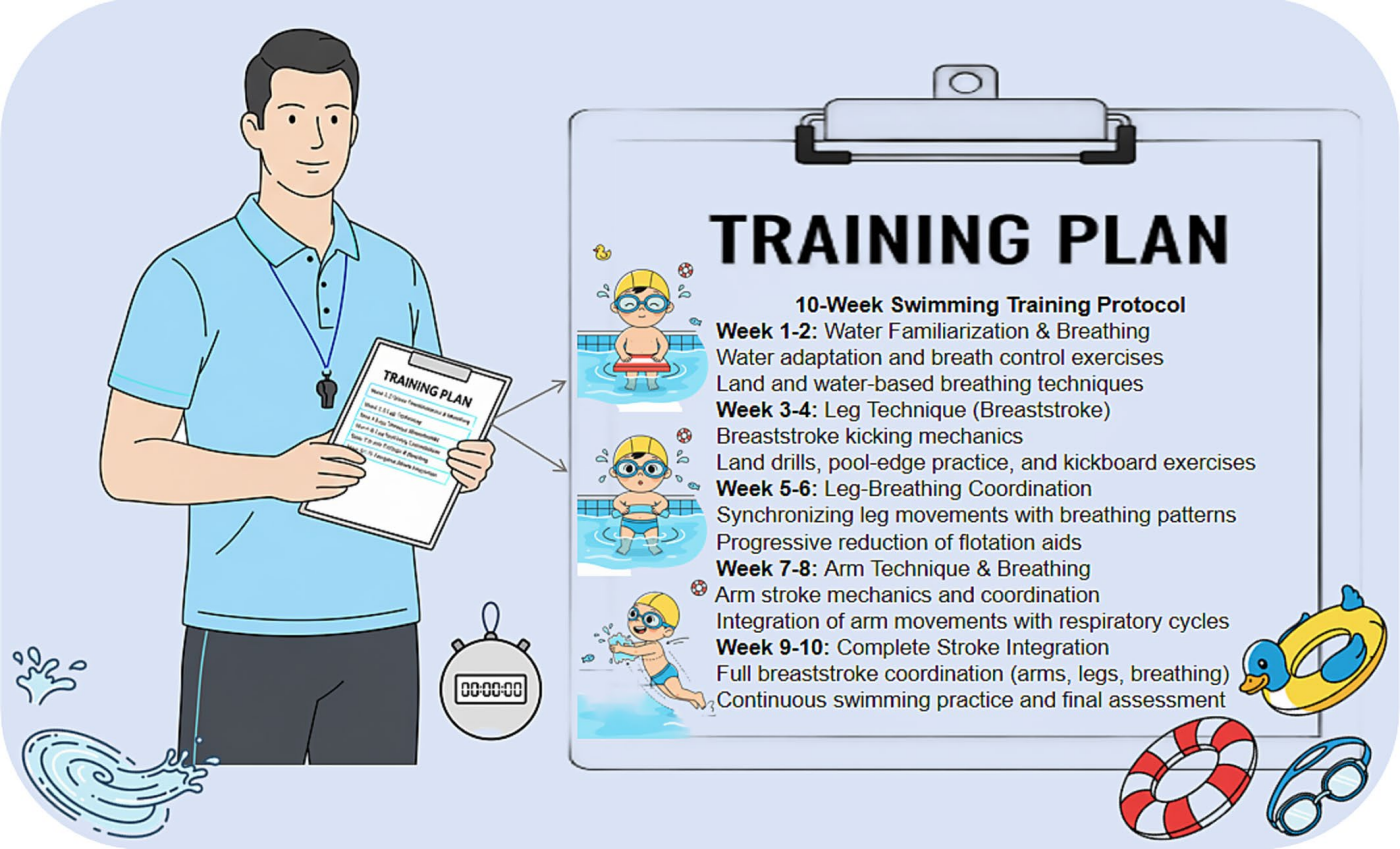
Schematic representation of the 10 week progressive breaststroke training protocol.

A 10-week breaststroke swimming program was implemented, consisting of 20 sessions (60 min each, twice weekly) in a temperature-controlled pool. Experienced coaches delivered a standardized curriculum designed with progressive complexity and intensity escalation from low to moderate-high levels across five developmental phases. To enhance reproducibility, the intervention was manualized with a standardized session template (warm-up, skill practice, and cool-down), phase-specific objectives, and predefined coaching cues. Pool conditions (e.g., water temperature, equipment, and session schedule) were kept consistent across sessions, and all sessions were delivered by the same coaching team following a briefing and rehearsal based on the manual. The selected dose (10 weeks, twice weekly, 60 min/session; 20 sessions in total) was intended to provide repeated, task-specific practice to support motor learning while remaining feasible for school-aged children and families. Session adherence was documented using a checklist, and any protocol deviations (if applicable) were recorded to support intervention fidelity and replicability. This protocol description is reported in detail to support reproducibility and intervention fidelity rather than to imply that all observed pre-post changes can be attributed solely to the program (Figure 2).

**Figure 3:**
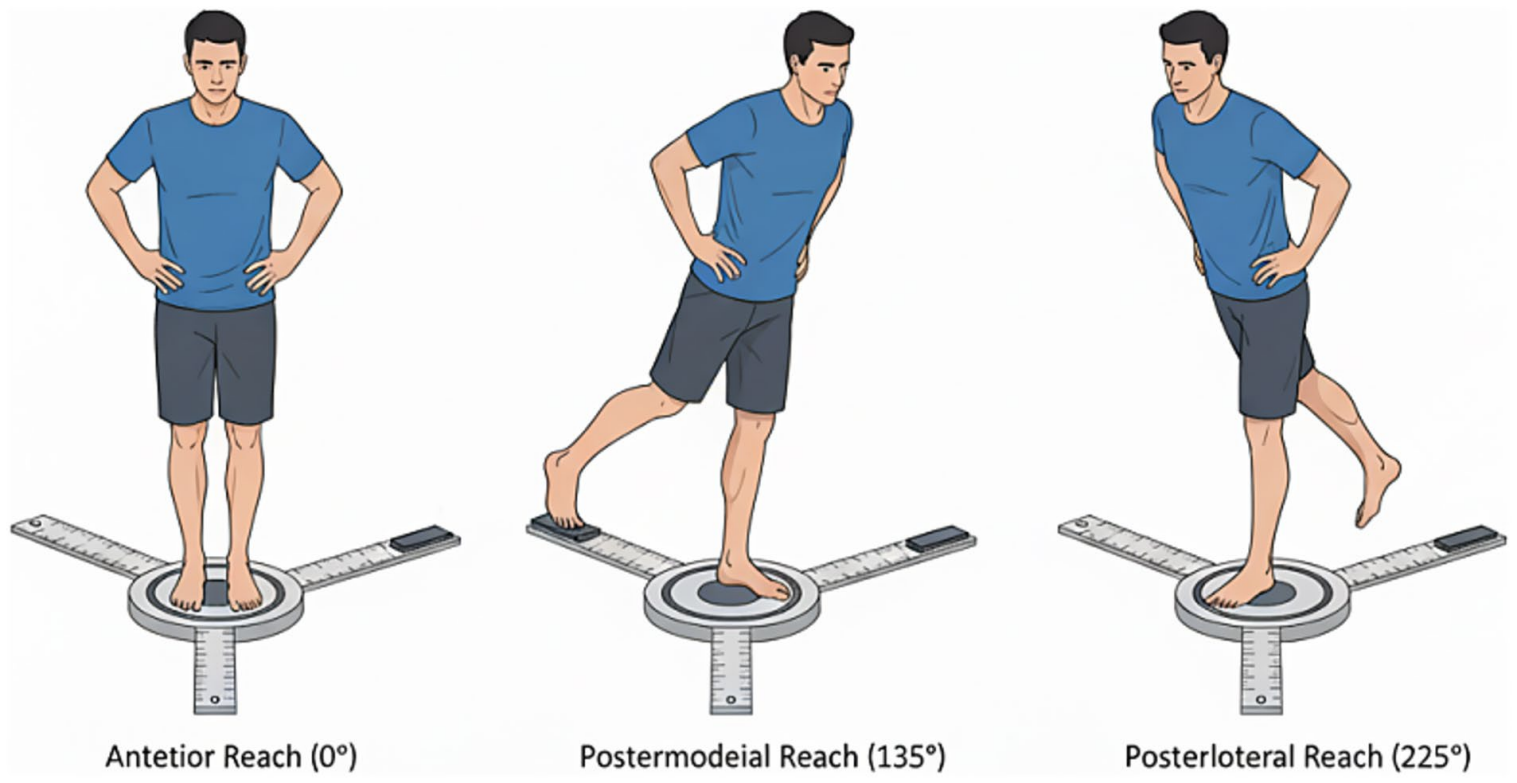
Y-balance test lower quarter (YBT-LQ) testing procedures.

Phase 1 (Week 1): Aquatic Adaptation. This initial phase aimed to establish water comfort and confidence. Participants performed breathing control exercises on land (inhalation-exhalation patterns) before transitioning to water-based activities including breath-holding and bubble production drills. The primary objective was developing voluntary facial submersion and controlled underwater exhalation capabilities (Figure 3).

Phase 2 (Weeks 2–3): Lower Limb Mechanics. Training concentrated on breaststroke kicking fundamentals. Participants learned the sequential leg movement pattern (knee flexion, foot positioning, explosive extension, and streamlined recovery) through land-based modeling, pool-edge supported practice, and kickboard-assisted prone drills. This phase established proper propulsive leg technique foundational to breaststroke swimming.

Phase 3 (Weeks 4–5): Leg-Respiration Integration. The focus shifted to coordinating leg movements with breathing cycles. Using flotation devices initially for support, participants practiced rhythmic kicking synchronized with inhalation-exhalation patterns. Assistance was systematically withdrawn as participants demonstrated competence in maintaining coordinated leg-breathing sequences during independent floating.

Phase 4 (Weeks 6–8): Upper Limb Technique. Arm stroke mechanics and arm-breathing synchronization were introduced through a similar pedagogical sequence: land-based instruction, resistance familiarization via pool-edge drills, and full immersion practice. Participants learned to coordinate propulsive arm movements with breathing phases, completing the respiratory-propulsion cycle for upper body technique.

Phase 5 (Weeks 9–10): Full Stroke Synthesis. The final phase integrated all components into complete breaststroke execution. Participants practiced continuous swimming with coordinated arm-leg-breathing sequences, refining technique fluency and building endurance. Session 20 included a performance assessment, with most participants demonstrating proficient whole-stroke technique and several achieving sustained swimming distances of 25 + meters.

### 2.5 Outcome measures

#### 2.5.1 Movement assessment battery for children-second edition (MABC-2)

The MABC-2 is a standardized assessment tool designed to identify and describe motor performance impairments in children and adolescents aged 3–16 years. The performance test comprises three age bands (3–6, 7–10, and 11–16 years), with eight tasks per band divided into three domains: Manual Dexterity, Aiming and Catching, and Balance. For this study, Age Band 2 (7–10 years) was administered.

Raw scores for each task were converted to standard scores (mean *=* 10, standard deviatio*n =* 3) according to age-adjusted norms. The sum of standard scores across all eight items yielded the Total Test Score (TTS), which was then converted to percentile ranks for interpretation (Schulz et al., 2011). Scores at or below the 5th percentile indicate definite motor impairment, scores between the 6th and 15th percentiles suggest borderline motor impairment, and scores above the 15th percentile reflect typical motor development (Wuang et al., 2012).

The MABC-2 has demonstrated high reliability and validity in children with and without motor impairments. Test–retest reliability for the total score has shown excellent intraclass correlation coefficients (ICC = 0.97), with internal consistency (Cronbach’s *α* = 0.90). The minimal detectable change (MDC) ranges from 0.28 to 5.76 points, providing reference values for determining clinically meaningful changes in individual children (Hua et al., 2013). In the present study, the MABC-2 served two purposes: first, as the school-based motor screening instrument used to identify children at risk for DCD; and second, as an outcome measure used to describe pre-post change in motor competence.

#### 2.5.2 Y-balance test-lower quarter (YBT-LQ)

The YBT-LQ is a reliable and valid assessment tool used to evaluate dynamic balance, proprioception, and functional movement patterns. The test requires participants to stand on one leg while reaching as far as possible with the contralateral leg in three directions: anterior (ANT), posteromedial (PM), and posterolateral (PL), forming a Y-shape (Shaffer et al., 2013).

Participants performed the test barefoot. Reach distances were measured in centimeters for each direction and subsequently normalized to leg length (expressed as percentage of leg length). Leg length was measured from the anterior superior iliac spine to the most distal aspect of the medial malleolus with participants in supine position. A composite score was calculated by summing the three reach distances for each leg and dividing by three times the leg length, then multiplying by 100.

The YBT-LQ has demonstrated excellent reliability with ICC values ranging from 0.85 to 0.99 for test–retest and inter-rater reliability (Smith et al., 2017; Powden et al., 2019). The test shows high reliability over time and between multiple raters, confirming its ability to accurately measure dynamic neuromuscular control. Normalized maximal reach distances for all three directions and composite scores have been established for healthy youth aged 10–17 years, providing age- and sex-specific reference values for comparison. The YBT-LQ was included as a complementary outcome measure to capture dynamic postural control and lower-extremity reach performance, and it was not used as part of participant screening or diagnostic classification.

Both the MABC-2 and YBT-LQ assessments were administered by trained examiners at pre-intervention (baseline) and post-intervention (after 10 weeks) time points. All assessments followed standardized procedures as outlined in their respective test manuals to ensure consistency and reliability of measurements. To reduce measurement bias, the same standardized instructions, scoring procedures, and test sequence were used at both time points wherever possible.

### 2.6 Statistical analysis

As this was a single-group pre-post design, all analyses focused on within-subject change from baseline to post-intervention rather than on between-group differences. The primary analytic objective was to characterize pre-post change in MABC-2 Total Test Score and YBT-LQ performance from T0 to T1, whereas changes in individual MABC-2 subdomains and directional YBT-LQ reach distances were treated as secondary exploratory outcomes. All statistical analyses were performed using R software (Version 4.3.1, R Foundation for Statistical Computing, Vienna, Austria). Descriptive statistics were calculated for all outcome variables, with continuous data presented as mean ± standard deviation (SD) or median with interquartile range (IQR), depending on the distribution of the data. Categorical variables were expressed as frequencies and percentages. Prior to conducting inferential analyses, the normality of data distribution was assessed using the Shapiro–Wilk test, which is recommended for sample sizes less than 50 (Ghasemi and Zahediasl, 2012). Visual inspection of Q-Q plots was also performed to evaluate normality assumptions.

For normally distributed continuous variables, paired-samples t-tests were used to compare pre- and post-intervention scores for both the MABC-2 and YBT-LQ measurements (Dwivedi et al., 2017). For these analyses, effect sizes were reported as Cohen’s dz. for paired samples, calculated on the basis of the standardized mean difference in pre-post change scores; values of approximately 0.20, 0.50, and 0.80 were interpreted as small, medium, and large effects, respectively. For variables that violated normality assumptions, the Wilcoxon signed-rank test was used. For Wilcoxon analyses, effect sizes were reported as r = Z/√N, where values of approximately 0.10, 0.30, and 0.50 were interpreted as small, medium, and large effects, respectively. Because all 14 enrolled participants completed both assessments, no imputation for missing data was required.

The alpha level for statistical significance was set at *p* < 0.05 (two-tailed) for all analyses. Because multiple related outcomes were tested, multiplicity was addressed using the Holm-Bonferroni procedure, applied separately to the four MABC-2 outcomes and the six YBT-LQ directional outcomes in order to control the family-wise error rate while preserving greater power than a simple Bonferroni correction. Both unadjusted *p* values and Holm-adjusted p values were reported for transparency. Because each endpoint involved only one planned pre-post comparison, no additional *post hoc* pairwise tests were required. To provide comprehensive information about the precision and clinical relevance of findings, 95% confidence intervals (CIs) were calculated for all effect estimates (Gardner and Altman, 1986). The 95% CI represents the range within which the true population parameter is expected to fall in 95% of repeated samples. Statistical assumptions were verified for each analysis, and any violations are reported with the corresponding results. Given the absence of a control group, the statistical analyses were intended to quantify the magnitude and direction of pre-post change within participants rather than to support causal inference regarding intervention efficacy. Accordingly, observed changes were interpreted cautiously because repeated testing may also introduce familiarization or practice effects.

## 3 Results

All 14 children at risk for DCD completed the 10-week aquatic-based breaststroke swimming intervention and both pre- and post-intervention assessments. No adverse events or dropouts were reported, resulting in a 100% retention rate.

### 3.1 Motor competence outcomes (MABC-2)

**Figure 4:**
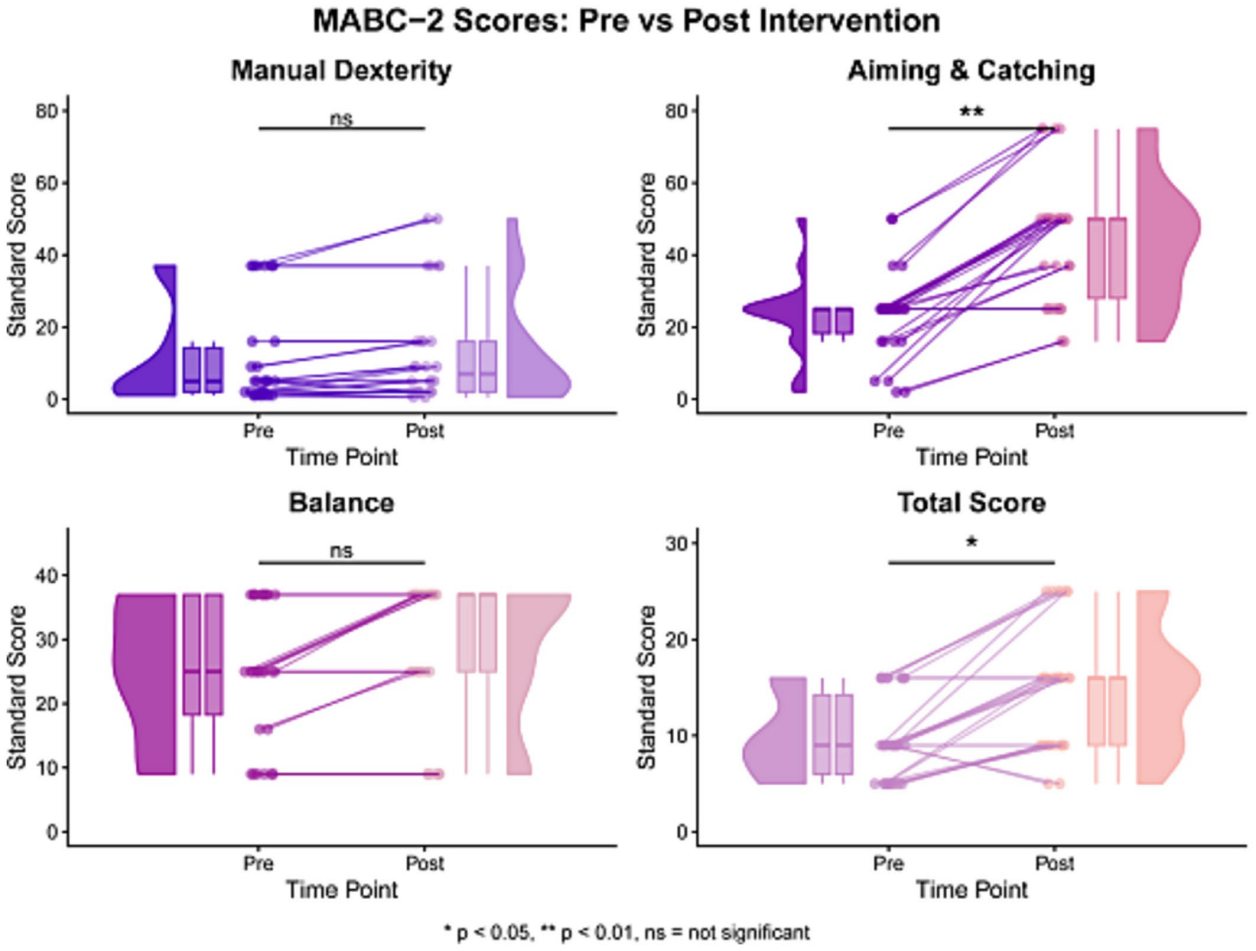
Distribution and individual changes in MABC-2 performance pre- to post-intervention. MABC-2 standardized scores before and after intervention. Wilcoxon signed-rank tests. *n =* 14.

Pre-post changes in motor competence are presented in Table 2 and Figure 4. MABC-2 Total Test Score increased from baseline (median 9, IQR 8.25) to post-intervention (median 16, IQR 7), with a median change of 5.50. This change remained statistically significant after Holm adjustment (unadjusted *p =* 0.007, adjusted *p =* 0.021) and was accompanied by a large effect size (r = 0.72).

**Table 2:** Pre-post changes in MABC-2 scores, effect sizes, and Holm-adjusted *p* values following the aquatic-based swimming intervention (*n =* 14).

Assessment	Dimension/Direction	Pre-intervention	Post-intervention	Change	Effect size (r)	Unadjusted *p*	Holm-adjusted *p*
MABC-2	Manual Dexterity	5 (12.25)	7 (14)	0.5	0.52	0.050	0.100
	Aiming and Catching	25 (6.75)	50 (22)	25.00	0.78	0.004	0.014
	Balance	25 (18.75)	37 (12)	0.00	0.45	0.089	0.100
	Total Score	9 (8.25)	16 (7)	5.50	0.72	0.007	0.021

Data are presented as median (interquartile range, IQR). Change represents the median change (post–pre). Unadjusted p values were calculated using the Wilcoxon signed-rank test. Holm-adjusted p values were used to account for multiple comparisons across the four MABC-2 outcomes. Effect size is reported as r = Z/√N, with values of approximately 0.10, 0.30, and 0.50 indicating small, medium, and large effects, respectively. For MABC-2, higher scores indicate better motor competence.

Analysis of MABC-2 subdomain scores revealed differential improvements across motor skill categories. Manual Dexterity scores improved from baseline (median 5, IQR 12.25) to post-intervention (median 7, IQR 14), with a median change of 0.5 (unadjusted *p =* 0.050, adjusted *p =* 0.100; r = 0.52), though this approached but did not reach statistical significance. The Aiming and Catching subdomain demonstrated significant improvement from baseline (median 25, IQR 6.75) to post-intervention (median 50, IQR 22), with a median change of 25.00 (unadjusted *p =* 0.004, adjusted *p =* 0.014; r = 0.78), representing substantial enhancement in ball skills and hand-eye coordination. The Balance subdomain showed improvement from baseline (median 25, IQR 18.75) to post-intervention (median 37, IQR 12), though the median change of 0.00 did not reach statistical significance (unadjusted *p =* 0.089, adjusted *p =* 0.100; r = 0.45), suggesting high inter-individual variability in balance responses. Individual response patterns demonstrated in Figure 4 revealed considerable heterogeneity in individual response patterns across participants.

Taken together, the MABC-2 results suggest that the clearest pre-post changes were observed in overall motor competence and the aiming-and-catching domain. The differing patterns across subdomains indicate that not all aspects of motor performance changed to the same extent, and these findings should therefore be interpreted as domain-specific exploratory signals in children at risk for DCD.

### 3.2 Dynamic balance outcomes (Y-balance test)

**Figure 5:**
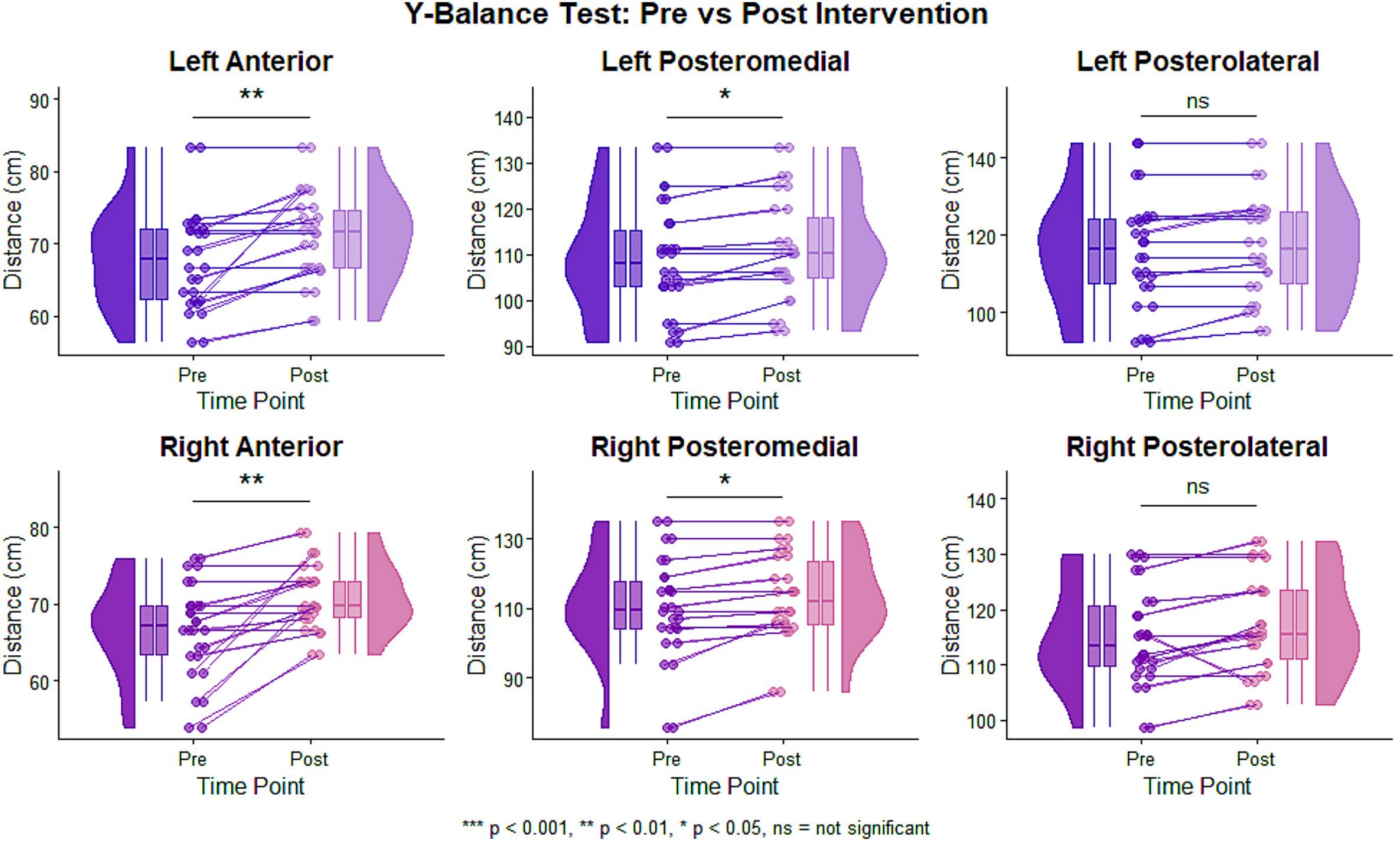
Distribution and individual changes in Y-balance test performance pre- to post-intervention. Pre- and post-intervention Y-balance test reach distances. Purple: left leg; pink: right leg (*n =* 14).

Pre-post changes in dynamic balance are presented in Table 3 and Figure 5. Before multiplicity adjustment, five of the six YBT-LQ reach directions showed significant improvement; after Holm correction, four directions remained statistically significant, namely left anterior, left posteromedial, right anterior, and right posteromedial reach. Effect sizes for these retained findings were in the medium-to-large range (Cohen’s dz. = 0.77–0.85).

**Table 3:** Pre-post changes in Y-balance test lower quarter (YBT-LQ) reach distances, effect sizes, and Holm-adjusted *p-*values following the aquatic-based swimming intervention (*n =* 14).

Assessment	Dimension/Direction	Pre-intervention	Post-intervention	Change (95% CI)	Effect size (Cohen’s dz)	Unadjusted *p*	Holm-adjusted *p*
Y-Balance Test	Left Anterior	67.82 ± 6.98	71.07 ± 6.31	3.26 [0.80, 5.71]	0.77	0.013	0.045
	Left Posteromedial	108.90 ± 12.35	111.06 ± 11.84	2.16 [0.59, 3.72]	0.79	0.011	0.045
	Left Posterolateral	115.58 ± 14.82	117.18 ± 13.99	1.60 [0.19, 3.01]	0.66	0.029	0.058
	Right Anterior	66.64 ± 6.30	70.82 ± 4.32	4.18 [1.31, 7.05]	0.84	0.008	0.045
	Right Posteromedial	110.19 ± 8.04	113.49 ± 12.94	3.30 [1.04, 5.55]	0.85	0.008	0.045
	Right Posterolateral	115.20 ± 9.24	117.37 ± 9.14	2.17 [−0.09, 4.43]	0.55	0.059	0.059

Data are presented as mean ± standard deviation (SD). Change represents the mean change (post–pre) with 95% confidence intervals (CIs). Unadjusted *p-*values were calculated using paired-samples t tests. Holm-adjusted p values were used to account for multiple comparisons across the six YBT-LQ directional outcomes. Effect size is reported as Cohen’s d for paired samples, with values of approximately 0.20, 0.50, and 0.80 indicating small, medium, and large effects, respectively. For the YBT-LQ, greater reach distances (cm) indicate better dynamic balance performance.

For the left leg, all three directions improved at the unadjusted level. Left Anterior reach distance increased from 67.82 ± 6.98 cm at baseline to 71.07 ± 6.31 cm post-intervention, representing a mean change of 3.26 cm (95% CI: 0.80–5.71; unadjusted *p =* 0.013, adjusted *p =* 0.045; Cohen’s dz. = 0.77). Left Posteromedial reach distance increased from 108.90 ± 12.35 cm to 111.06 ± 11.84 cm, with a mean change of 2.16 cm (95% CI: 0.59–3.72; unadjusted *p =* 0.011, adjusted *p =* 0.045; Cohen’s dz. = 0.79). Left Posterolateral reach distance also improved numerically from 115.58 ± 14.82 cm to 117.18 ± 13.99 cm, with a mean change of 1.60 cm (95% CI: 0.19–3.01; unadjusted *p =* 0.029), but this effect did not remain significant after Holm correction (adjusted *p =* 0.058), despite a moderate effect size (Cohen’s dz. = 0.66).

For the right leg, significant improvement was observed in the anterior and posteromedial directions both before and after multiplicity adjustment. Right Anterior reach distance increased from 66.64 ± 6.30 cm to 70.82 ± 4.32 cm (mean change 4.18 cm, 95% CI: 1.31–7.05; unadjusted *p =* 0.008, adjusted *p =* 0.045; Cohen’s dz. = 0.84). Right Posteromedial reach distance improved from 110.19 ± 8.04 cm to 113.49 ± 12.94 cm (mean change 3.30 cm, 95% CI: 1.04–5.55; unadjusted *p =* 0.008, adjusted *p =* 0.045; Cohen’s dz. = 0.85). Right Posterolateral reach distance increased from 115.20 ± 9.24 cm to 117.37 ± 9.14 cm (mean change 2.17 cm, 95% CI: −0.09-4.43), but this change was not statistically significant (unadjusted *p =* 0.059, adjusted *p =* 0.059; Cohen’s dz. = 0.55). Individual response patterns in Figure 5 suggested relatively consistent gains in the anterior and posteromedial directions.

These findings indicate pre-post improvements in dynamic balance in children at risk for DCD, with the bilateral pattern of change suggesting that the observed gains were not limited to one lower extremity.

## 4 Discussion

### 4.1 Main findings and interpretation

This single-group pre-post study described changes in motor competence and dynamic balance following a 10-week aquatic-based breaststroke swimming program in children at risk for DCD. The clearest pre-post changes were observed in MABC-2 Total Test Score and in the Aiming and Catching subdomain, whereas Manual Dexterity and the MABC-2 Balance subdomain did not show statistically robust changes. For the YBT-LQ, improvement was evident in several reach directions, but after Holm correction the most stable findings were concentrated in the anterior and posteromedial directions of both lower extremities rather than across all directions. Taken together, these findings suggest a pattern of domain-specific change rather than uniform improvement across all motor outcomes. The program also appeared feasible within the context of the present study, as reflected by full retention and the absence of adverse events.

The increase in MABC-2 Total Test Score may indicate that repeated aquatic motor practice was associated with improvement in overall motor performance at the group level. This interpretation is broadly consistent with the wider rehabilitation and motor-intervention literature, which has reported beneficial short-term changes following task-oriented and aquatic-based practice in children with motor difficulties (Hillier et al., 2010; Smits-Engelsman et al., 2013). The aquatic environment may help some children perform repeated whole-body actions with greater confidence and movement stability (Gao et al., 2024); however, these proposed mechanisms remain hypothetical in the present study because no direct measures of sensory integration, motor planning, or neuromuscular adaptation were collected.

### 4.2 Domain-specific motor competence changes

A notable feature of the MABC-2 findings was that change was not uniform across subdomains. The largest shift was observed in Aiming and Catching, whereas Manual Dexterity and Balance showed smaller and less stable changes. One possible interpretation is that the swimming program provided repeated opportunities for temporal coordination, limb sequencing, and visually guided action, all of which may be more closely related to the demands captured by the Aiming and Catching domain than to fine hand manipulation tasks. This interpretation is consistent with previous work suggesting that motor interventions in DCD often influence some motor domains more clearly than others, depending on the specificity of the practiced tasks and the transfer demands of the outcome measure. However, because the intervention did not include a direct ball-skill component, the present findings should not be interpreted as evidence of a specific transfer mechanism; rather, they suggest that some aspects of whole-body coordination may have generalized more readily than others.

The absence of clear statistical change in Manual Dexterity and the MABC-2 Balance subdomain is also important for interpretation. These findings suggest that the observed pattern of pre-post change did not suggest comparable change across all domains of motor competence to the same extent, particularly outcomes that depend more strongly on fine hand control or on task demands that differ from those practiced in the intervention. In addition, individual response patterns in Figure 4 indicate appreciable between-participant variability, which may reflect heterogeneity in baseline motor profiles, responsiveness to practice, or day-to-day performance fluctuation (Biotteau et al., 2016b; Allen and Casey, 2017). Accordingly, the present results do not support the view that aquatic practice is broadly effective across all components of motor competence; instead, they suggest that future controlled studies should examine which children and which motor domains are most likely to benefit from this type of training.

### 4.3 Dynamic balance outcomes and potential mechanisms

The YBT-LQ findings suggest that the most consistent balance-related changes were concentrated in the anterior and posteromedial directions of both lower extremities, whereas the posterolateral directions showed smaller and less stable changes after multiplicity adjustment. This directional pattern is relevant because it suggests that the observed balance-related changes were selective rather than global. One possible interpretation is that repeated breaststroke practice required continuous control of body alignment, lower-limb coordination, and trunk stabilization, which may have transferred more readily to some dynamic balance directions than to others. More broadly, the aquatic environment has often been discussed as a context that provides rich multisensory input through water resistance, hydrostatic pressure, and altered gravitational loading (Prins and Cutner, 1999; Getz et al., 2007; Pratt et al., 2024). These features may support postural regulation and movement calibration during practice; however, in the present study they should be regarded as plausible explanatory hypotheses rather than demonstrated mechanisms.

The bilateral pattern of change observed in our study is noteworthy. The presence of changes in both lower extremities may reflect generalized adaptations in postural control and movement coordination rather than effects confined to one limb. This bilateral pattern may have relevance for real-world activities requiring coordinated bilateral limb control (Silva et al., 2020). However, the non-significant improvement in right posterolateral reach (*p =* 0.059) suggests that certain movement directions may require more targeted practice or longer intervention duration to achieve meaningful changes.

Several neurophysiological mechanisms may help explain these pre-post changes, although they cannot be directly tested in the present study. First, buoyancy and water support may allow children with poor coordination to rehearse movement sequences with reduced fear of failure and reduced postural threat, thereby increasing the quantity and continuity of successful practice (Licari et al., 2015). Second, the continuous resistance of water may provide dense proprioceptive and tactile input throughout the movement trajectory, which could assist movement regulation and error detection during practice (Becker and Cole, 2011). Third, the cyclical and rhythmically structured nature of breaststroke may place demands on timing, sequencing, and inter-limb coordination that are relevant to some aspects of motor control in children at risk for DCD (Gorter and Currie, 2013). These possibilities are theoretically coherent with current accounts of DCD-related motor difficulties, but they remain speculative here and should be tested directly in future studies using process-oriented measures.

### 4.4 Feasibility and practical relevance

A practical strength of the present study was the high intervention adherence. All enrolled children completed the 10-week program and no adverse events were reported, suggesting that this manualized swimming format was feasible and acceptable within the specific context in which it was delivered. This observation is consistent with broader aquatic-intervention literature reporting good participation and tolerability in pediatric disability settings (Novak et al., 2020; Ogonowska-Slodownik et al., 2025).

The feasibility findings of the present study are context-dependent. Because the intervention was delivered in a temperature-controlled pool by trained coaches and under relatively close supervision, the results should be interpreted as evidence of feasibility under structured conditions rather than as support for broad implementation across all settings. In schools or communities without structured swimming facilities, appropriate safety procedures, or stable scheduling capacity, the present protocol may be difficult to implement, and alternative land-based or lower-resource motor interventions may be more practical.

The median pre-post change in MABC-2 Total Test Score was 5.50 points, close to the upper bound of the published MDC range (0.28–5.76 points), suggesting that the observed change may exceed measurement error and may be meaningful from practical and rehabilitation-oriented perspectives. Such a change could plausibly be relevant to school- and daily-life motor participation. However, because functional impairment and participation were not directly measured using a separate standardized instrument, and because a universally accepted MCID or responder threshold for MABC-2 intervention studies was not available, this interpretation should remain cautious.

### 4.5 Limitations

Several limitations should be emphasized. First, the study used a single-group pre-post design without a control condition; consequently, the observed changes cannot be attributed specifically to the swimming program and may partly reflect maturation, repeated-testing effects, regression to the mean, or other contextual influences. Second, the sample was small, recruited from a single school, and initially identified through teacher-based pre-screening before formal MABC-2 assessment, which may have preferentially captured children with more visible coordination problems and thereby introduced selection bias. In addition, the study was conducted in a single school serving its surrounding community in Chengdu, a large and highly urbanized city with a complex population background. Within the broader Sichuan context, where population composition is relatively diverse, the source school may have drawn from a socially and culturally mixed community rather than a narrowly homogeneous population. These contextual features may have influenced family participation, program adherence, and the practical feasibility of the intervention. However, because the sample was still recruited from only one school and no direct demographic data on cultural or ethnic background were collected, these contextual considerations should be viewed as setting-related factors rather than evidence of broad population representativeness. Third, participants were classified as children at risk for DCD on the basis of MABC-2 performance rather than a formal DSM-5 diagnostic process, while functional impairment in everyday activities was not assessed using a separate standardized measure and co-occurring neurodevelopmental conditions were not evaluated with formal diagnostic instruments. These factors limit direct generalization to clinically diagnosed DCD populations, whose motor profiles and intervention responses may differ because of more clearly documented functional impairment, greater clinical heterogeneity, and the possible presence of additional co-occurring conditions. Fourth, although multiplicity adjustment and effect-size reporting were added to improve statistical transparency, several secondary outcomes remained exploratory and the study was not separately powered for all subdomain-level or directional analyses. Fifth, outcome assessment was limited to immediate post-intervention change, so the durability of the observed pattern remains unknown. Future research should incorporate control conditions, larger samples, extended follow-up periods, and functional outcome measures beyond standardized motor assessments to more comprehensively evaluate potential intervention effects (Lingam et al., 2012). Additionally, investigation of optimal intervention parameters and identification of treatment response predictors would inform development of personalized protocols (Caçola et al., 2016).

Despite these limitations, the present study provides preliminary evidence that a structured aquatic-based swimming program may be associated with favorable short-term changes in selected aspects of motor competence and dynamic balance in children at risk for DCD. The findings are most appropriately interpreted as a feasibility-informed signal of potential benefit rather than as proof of intervention efficacy. Within that framework, the study supports further controlled evaluation of structured swimming as a complementary movement-based option for children with coordination difficulties. Future trials should determine whether the observed motor changes are reproducible, whether they extend to functional participation and psychosocial outcomes, and which children are most likely to benefit.

## 5 Conclusion

In this single-group pre-post study, children at risk for DCD demonstrated notable improvements in selected measures of motor competence and dynamic balance throughout the 10-week investigation period, with the most significant enhancements recorded in the MABC-2 Total Test Score, aiming and catching tasks, and multiple YBT-LQ reach directions.

These findings further indicate the program’s viability within this participant cohort, evidenced by complete participant retention and the absence of adverse events during the implementation of a standardized, progressive twice-weekly curriculum administered by qualified instructors in a climate-controlled aquatic environment.

In practical applications, this approach may merit inclusion as an adjunctive component within school- and community-based physical activity programs where aquatic facilities are already accessible; nevertheless, its potential effectiveness and replicability necessitate verification through controlled investigations before widespread implementation recommendations can be established. The organized, non-daily structure may also present practical advantages in settings with existing pool availability; however, the broader applicability of this implementation model requires validation through larger and more diverse participant populations.

Subsequent investigations employing controlled methodologies, larger sample sizes, and longitudinal assessments are necessary to evaluate the sustainability of these observed improvements, identify responsive predictors, and optimize implementation protocols.

## Data Availability

The raw data supporting the conclusions of this article will be made available by the authors, without undue reservation.
