# Supplementary material for: Effects of swimming intervention on motor competence and dynamic balance in children at risk for developmental coordination disorder: a pre-post intervention study
Source: Front Psychol. 2026 May 4;17:1815930. doi: 10.3389/fpsyg.2026.1815930 (PMC13180587; doi:10.3389/fpsyg.2026.1815930)
Supplement: Supplementary file 1 [file Supplementary_file_1.docx]

**Supplementary**


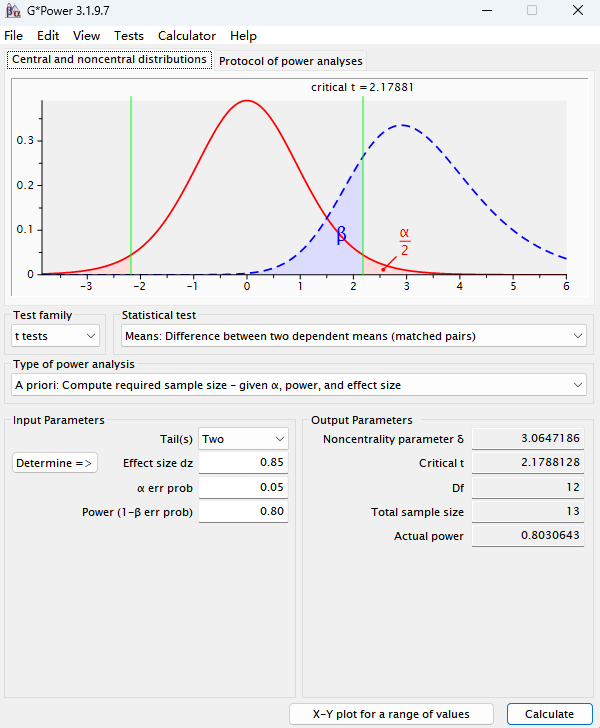


**Figure S1. A priori power analysis for sample size determination.**

G*Power 3.1.9.7 was used to calculate the required sample size for a paired t-test (two-tailed). Based on an expected large effect size (dz = 0.85), alpha level of 0.05, and desired power of 0.80, the analysis indicated that a minimum of 13 participants would be required. The actual achieved power with 13 participants was 0.8030643.


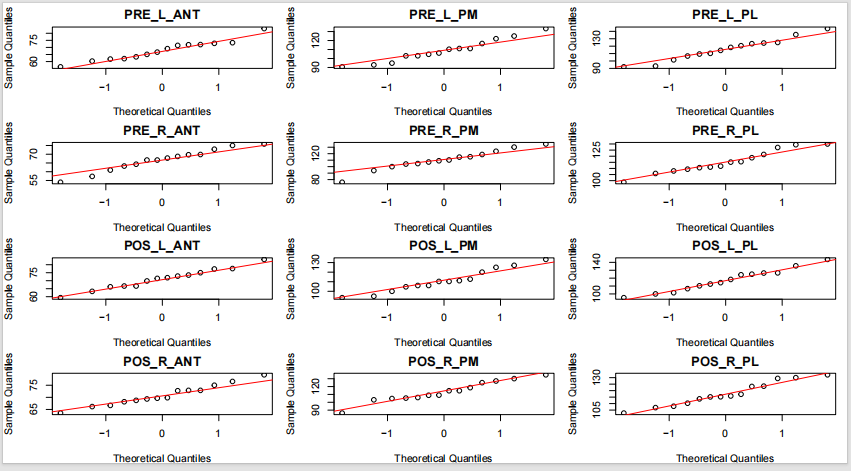


**Figure S2. Quantile-quantile plots showing normal distribution of pre- and post-intervention Y-Balance Test scores for all directions**

Quantile-quantile plots demonstrate the normal distribution of pre-intervention (PRE) and post-intervention (POS) Y-Balance Test scores for left (L) and right (R) limbs across three reach directions: anterior (ANT), posteromedial (PM), and posterolateral (PL). Data points closely follow the theoretical normal distribution line (red), indicating that the assumption of normality is satisfied for parametric statistical analysis.

**
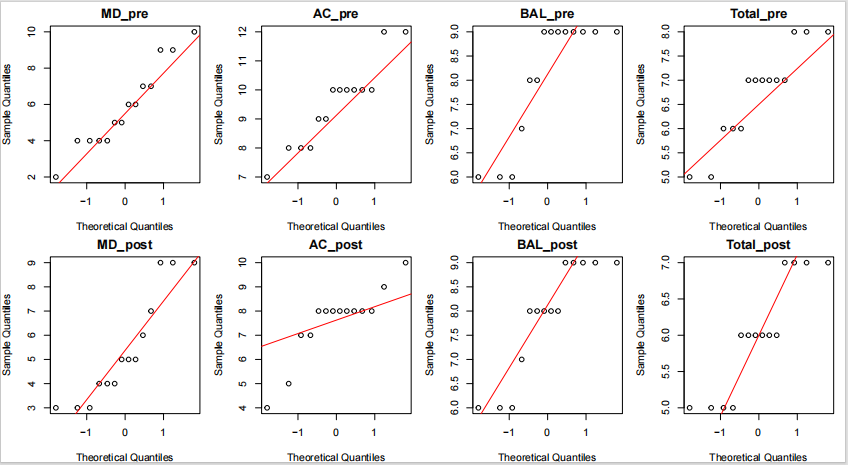
**

**Figure S3. Quantile-quantile plots showing normal distribution of pre- and post-intervention MABC-2 scores (Manual Dexterity, Aiming & Catching, Balance, and Total Test Score)**

Quantile-quantile plots demonstrate the normal distribution of pre-intervention (pre) and post-intervention (post) MABC-2 scores across three components: Manual Dexterity (MD), Aiming & Catching (AC), Balance (BAL), and Total Test Score. Data points closely follow the theoretical normal distribution line (red), confirming the appropriateness of parametric testing (paired t-tests).
